# Dr. J. E. Garretson, Dentist or Surgeon?

**Published:** 1904-12

**Authors:** 


### DR. J. E. GARRETSON, DENTIST OR SURGEON?

The following paragraph taken from a paper on "The Danger of allowing Warts and Moles to remain," etc., by Dr. W. W. Keen, read before the American Medical Association, at Atlantic City, in June last, is one of those peculiar discriminations dealt in by some medical men whenever they have occasion to allude to the work of another member of the same profession, provided he happens to practise a specialty such as dentistry. "He is a dentist," and that of course settles his place in the domain of medicine. The quotation is as follows:

"*History.*—Mrs. F., aged about sixty, was first seen in consultation with Dr. Rhoads, February 7, 1894. Ever since she can remember she had a wart on the right ankle, just below the inner malleolus. In 1889, five years ago, this began to enlarge, and some time afterwards the late Dr. J. E. Garretson, a dentist, transfixed it with a pin and ligated it. It soon returned, and a year later was again pinned and ligated by her son, who was also a dentist. Recurrence again took place, and in July, 1893, the growth was again transfixed and ligated by Dr. Garretson. Ten months before the last operation the saphenous glands were observed to be enlarged. At the time of the last operation the saphenous tumor had grown to the size of a lady apple. A considerable amount of pigmentation also had appeared in the neighborhood of the original tumor, extending towards the toes."

The fact was that Dr. Garretson was a graduate of medicine, University of Pennsylvania, 1859, a professor of anatomy, a surgeon, and an author of world-wide celebrity. As far as the writer is aware, his practice of dentistry was limited, his tastes running in the direction of surgery, for the practice of which he was well qualified by education and experience.

The quotation seems to imply that the fact of the ligation being performed on two separate occasions by two dentists, it was not done according to the high standard of the surgeon. Why was it necessary to mention the specialty at all? It will be some generations hence before the feeling, "He is only a dentist," is eradicated from the minds of medical practitioners, who dwell in the narrow confines of their own specialty and are apparently oblivious to the fact that the world of intelligence progresses steadily towards a higher standard of thought and practice.

